# Nitrogen Metabolism during Pregnancy

**Published:** 1916-10

**Authors:** 


**Nitrogen Metabolism During Pregnancy.** Karl M. Wilson, Bull. Johns Hopkins Hosp., May, 1916. Several detail tables are presented. In two cases, for 10 and 7 days of the puerperium, respectively, the averages were:

| | | |
|---|---|---|
| Nitrogen intake | 11.34 Grams | 18.03 Grams |
| Nitrogen output | | |
| Urine | 10.91 | 15.96 |
| Faeces | .82 | 1.06 |
| Lochia | .49 | 3.40 |
| Milk | .70 | .32 |
| Total | 12.92 | 20.74 |

The nitrogen content of the ovum was estimated as follows: foetus 62.10, placenta 14.66, cord and membranes 2., amniotic liquid 0.19; total 78.95. In another case that stored 336.21 grams of nitrogen in 14½ weeks, the amount of stored nitrogen diverted to the development of the ovum was estimated as: foetus 64.5, placenta 11.73, membranes and cord 2., amniotic liquid 0.68, total 78.91. The following general conclusions are drawn:

1. In the perfectly normal pregnant woman, storage of nitrogen begins at a much earlier period than has hitherto been supposed; possibly the organism may acquire the capacity for storing nitrogen from the very beginning of the pregnancy.

2. In the early months this storage is far in excess of the actual needs of the developing ovum, and the excess must be added to the general maternal organism.

3. Storage of nitrogen continues throughout the entire duration of pregnancy, being most marked during the last few weeks, when the foetal needs are at a maximum.

4. The nitrogen stored is greatly in excess of the actual needs of the developing ovum, so that, apart from the amount needed for the hypertrophy and development of the genitalia and breasts, a large proportion of the nitrogen stored is added to the general maternal organism as "Restmaterial," though, concerning the form in which this reserve is stored, we are unable to make any positive statement. The nitrogen capital of the maternal organism is thus increased, though the reserve supply may possibly be entirely exhausted during the puerperium and period of lactation.

5. In the healthy woman, who goes through a normal pregnancy, the period of gestation does not necessarily represent a "sacrifice of the individual for the sake of the species," but may actually be a period of gain.

6. There is a relative increase in the percentage of urinary nitrogen excreted in the form of free amino-acids, though not necessarily an absolute increase in this form of nitrogen.

7. There is also a tendency for the percentage of ammonia nitrogen to become increased during the last weeks of pregnancy, although at other times during the pregnancy there is practically no variation from the percentages noted in non-pregnant individuals upon a similar diet.

